# Playing for keeps or just playing with emotion? Studying tilt and emotion regulation in video games

**DOI:** 10.3389/fpsyg.2024.1385242

**Published:** 2024-04-26

**Authors:** Sarah C. Cregan, Adam J. Toth, Mark J. Campbell

**Affiliations:** ^1^Department of Physical Education and Sport Sciences, Faculty of Education and Health Sciences, University of Limerick, Limerick, Ireland; ^2^Lero - Science Foundation Ireland Research Centre for Software, University of Limerick, Limerick, Ireland; ^3^Faculty of Education and Health Sciences, Health Research Institute, University of Limerick, Limerick, Ireland; ^4^Centre for Sport Leadership, Maties Sport, Stellenbosch University, Stellenbosch, South Africa

**Keywords:** competitive gaming, coping strategies, game genre, emotional control, choking, esports

## Abstract

**Introduction:**

In video gaming, tilt is thought to relate to poor emotional control and game performance. Despite widespread recognition of tilt in video gaming, there is a lack of research examining tilt empirically.

**Methods:**

One thousand and seven gamers took part in our online study examining gamers experience of tilt, the factors which contribute to and protect against tilt, and the emotion regulation strategies gamers employ to deal with tilt.

**Results:**

Gamers who reported playing for more competitive reasons, were at higher risk of experiencing tilt. Additional factors associated with an increased risk of experiencing tilt were increased anger and more hours spent playing. Protective factors against experiencing tilt were also identified, inclusive of a greater number of years gaming experience and engagement in adaptive emotion regulation strategies.

**Discussion:**

This study provides an important starting point for creating a better understanding of tilt in gaming, equipping us with new knowledge to better support gamers to improve their emotion regulation during game play performance

## Introduction

Video games are a highly popular activity, with an estimate of over 2.6 billion gamers worldwide (Pallavicini et al., 2021). Esports, which incorporates video games that are played competitively and professionally (Campbell et al., 2018), have also grown rapidly in popularity. In line with large prize pools for esports competitions, and viewership surpassing that of large sporting events such as the National Basketball Association Finals (Cranmer et al., 2021), there has been increased demand to better understand the health implications of esports and video games.

Researchers have predominantly been concerned with debating the benefits and disadvantages of video gaming. Researchers are increasingly recognizing the potential of videogames to provide cognitive, social, and health benefits. In relation to cognitive benefits, studies have shown that action video gamers demonstrate superior attentional control, task switching (Toth et al., 2020), information processing (Kowal et al., 2018) and working memory abilities (Moisala et al., 2017). Video games have also been shown to positively affect psychological well-being (Halbrook et al., 2019), induce positive emotions (Osmanovic and Pecchioni, 2016), mitigate symptoms of depression and anxiety (Kowal et al., 2021) and improve emotion regulation (Villani et al., 2018). Video games also play a role in digital therapies, with specifically designed games generally aiding in the diagnosis and symptom reduction of disorders such as attention deficit hyperactivity disorder (Peñuelas-Calvo et al., 2022) and improving both reading abilities and visual–auditory attentional shifting in children with dyslexia (Franceschini et al., 2017). The recognition of the social benefits of video games extends to research demonstrating that multiplayer and augmented reality games can reduce loneliness and mitigate stress, depression, and anxiety (Kowal et al., 2021; Pallavicini et al., 2022), facilitating pro social behavior (Wiederhold, 2021).

Turning to the purported negative effects of video games, researchers have debated the association between violent video games and aggression, an area of literature with much disagreement (Halbrook et al., 2019). Breuer et al. (2015) examined frustration and video games, acknowledging that competition in video games can be rewarding and enjoyable but also potentially a source of frustration when attempts to reach goals are blocked. Pzybylski et al. (2014) suggest that players can become frustrated if the challenge of the game is overly difficult and exceeds the players skill level, potentially rage quitting the game due to intense negative emotions, highlighting why game designers attempt to make games incrementally more challenging and skill match players (Pzybylski et al., 2014).

Research has also focused on problematic and excessive video game engagement. This excessive engagement has been labeled as Internet Gaming Disorder (IGD). IGD has been officially recognized by the American Psychiatric Association (2022) and is characterized by impaired control over gaming habits, gaming taking priority over other daily activities and continued engagement in gaming despite negative consequences (WHO, 2019). In people diagnosed with IGD, some researchers have found IGD to be linked to poor psychological wellbeing (Teng et al., 2020), general anxiety disorder (Wang et al., 2017) and reduced academic achievement (Hawi et al., 2018). Yen et al. (2017) examined Internet gaming disorder and emotion regulation. Emotion regulation relates to the process in which we influence, experience and express the emotions we have (Gross, 1998). Yen et al. (2017) found individuals with IGD to be more likely to engage in emotional suppression and less likely to engage in cognitive reappraisal.

The ability to regulate our emotions effectively is important for psychological wellbeing (Tasneem and Panwar, 2022), social relationships (Lopes et al., 2005), social functioning (English et al., 2012), and physical health (Song et al., 2015). Strategies to regulate emotion in research have been broadly categorized as either adaptive or maladaptive (Aldao et al., 2010). Maladaptive emotion regulation relates to the inability to flexibly respond to demands (Aldao et al., 2015) and can result in the unsuccessful reduction of negative emotions (Campbell-Sills et al., 2013). Examples of maladaptive emotion regulation include engaging in strategies to regulate emotions such as rumination or suppression (Navas-Casado et al., 2023). Contrastingly, adaptive emotion regulation requires the ability to flexibly alternate strategies depending on one’s contextual demands and goals (Campbell-Sills et al., 2013; Aldao et al., 2015). An example of adaptive emotion regulation includes cognitive reappraisal. Adaptive emotion regulation strategies are associated with better wellbeing, interpersonal functioning (Gross and John, 2003) and physical health (Song et al., 2015).

In traditional sport, researchers recognize the importance of adaptive emotion regulation in improving performance and alleviating occurrence of choking (Balk et al., 2013). In sport, choking relates to player underperformance in pressurized situations (Baumeister, 1984) despite having the necessary skillset and motivation to perform well (Hill et al., 2010; Gröpel and Mesagno, 2019). Choking not only negatively impacts performance, but also reduces executive control (Belletier et al., 2015). In traditional sport, there is mixed evidence regarding gender differences in experience of choking among athletes. In both Cohen-Zada et al. (2017) and Bühren et al. (2024) studies, researchers found males to be more susceptible to choking due to performance pressure. Contrastingly, Min (2022) found high level female athletes to underperform in pressurized competitive situations compared to males in segregated, but not mixed-gender competition.

The concept of choking in sport shares similarities with tilt in video gaming. The term tilt originates from the arcade game of pinball, where an individual would become frustrated and shake the pinball machine, causing the word “tilt” to appear on the screen and the game to be lost (Duncan, 2015). The term today is commonly employed in poker, video gaming and esports. Research in poker suggests that tilt occurs due to performance pressure in relation to potential monetary loss (Browne, 1989). Loses can induce negative emotions such as anger, frustration, and injustice, and potentially induce tilt (Browne, 1989; Palomäki et al., 2014). There is mixed evidence pertaining to the role of player experience in reducing experience of tilt (Palomäki et al., 2013, 2014), with researchers highlighting the potential role of player experience in dealing with monetary losses and employment of better emotion regulation strategies.

Despite recognition of tilt in video gaming, there is a lack of empirical research examining tilt in gaming. We currently do not know how gamers experience tilt, their emotion regulation strategies to deal with tilt and the factors that contribute to their experience of tilt. The purpose of the current study is to examine gamers’ experience of tilt and emotion regulation. By doing so, we seek to better understand the extent to which gamers experience tilt, identify factors that may reduce or exacerbate tilt and gain a better understanding about the emotion regulation strategies that impact gamers experience of tilt, to inform future interventions.

Firstly, given the recognition that motivation to perform well can impact choking in traditional sport (Baumeister, 1984; Toma, 2017; Gröpel and Mesagno, 2019), we hypothesize that there will be a difference in experience of tilt based on the players reason for playing. Secondly, given the mixed evidence on gender differences in choking under pressure in sport (Cohen-Zada et al., 2017; Min, 2022; Bühren et al., 2024), we hypothesize that there will be a difference in the experience of tilt due to gender. Lastly, we aimed to explore which factors would be most influential in explaining gamers’ experience of tilt.

## Methods

### Participant recruitment

A power analysis was conducted using G*Power version 3.1.9.7 (Faul et al., 2009) to determine the sample size needed to run a 2 way-ANOVA. The significance criterion was set at α = 0.05 and power = 0.80, requiring a minimum sample size of *N* = 128. A sample of *N* = 1958 participants completed an online questionnaire using the Qualtrics Survey Platform (Qualtrics, Provo, UT), with invitations to partake in the study circulated via gaming social media platforms and email recruitment. Eligible participants included anyone aged 18 years of age or older that currently played video games. Participants all provided written consent prior to partaking in the study. This research study was approved by the Faculty of Education and Health Sciences Research Ethics Committee at the University of Limerick [Study ID EHSREC 2023-06-25] and is in accordance with the Declaration of Helsinki.

### Procedure

In the first section of the questionnaire, participants provided demographic information pertaining to their age, gender, and the country they played video games in. Participants also reported the game genre they play most often, the number of years they had been gaming for and the hours they spend gaming per week. Participants were asked to select their main reason for playing videogames by selecting one of the following responses (i) for fun, (ii) to win or (iii) to improve.

Participants then rated 2 items, which were adapted from the Severity of Tilting Scale (Palomäki et al., 2014), and completed both the Behavioral Emotion Regulation Questionnaire (BERQ) (Kraaij and Garnefski, 2019) and the Sports Emotion Questionnaire (SEQ) (Jones et al., 2005) in relation to their experience of tilt. Details of the questionnaires are provided below.

### Materials

#### Items adapted from severity of tilting scale

As there is currently no available scale to measure tilt in gaming, 2 items were adapted from the four item Severity of Tilting Scale, designed by Palomäki et al. (2014) to measure tilt severity among poker players. To our knowledge, this is the first study to try quantifying gamers experience of tilt in video gaming. Two items from the Severity of Tilting scale were excluded as they were not deemed applicable for examining tilt in the context of video gaming. The two adapted items related to participants perception of both the *frequency* and *intensity* in which they experienced tilt. For our study, these items were rated on a five-point Likert scale and summed to obtain an individual’s total tilt score, with higher scores indicative of a greater experience of tilt.

#### Behavioral emotion regulation questionnaire

The behavioral emotion regulation questionnaire (Kraaij and Garnefski, 2019) assesses one’s behavioral strategies to regulate emotions in response to a particular stressful event/ situation. The BERQ consists of 20 items, each rated on a five-point Likert scale, and with four items attributed to 5 subscales. These subscales refer to 5 distinct regulation strategies inclusive of *Seeking Distraction*, *Withdrawal*, *Actively Approaching*, *Seeking Social Support*, and *Ignoring*. Higher total scores on a subscale indicate greater employment of a strategy, with an individual able to score a maximum of 20 on any subscale. Literature suggests that seeking distraction, actively approaching, and seeking social support are positive strategies to cope with stress whereas ignoring and withdrawing are negative coping strategies (Joormann and Stanton, 2016; Kraaij and Garnefski, 2019).

#### Sports emotion questionnaire

The sports emotion questionnaire (Jones et al. 2005) was created as a means of capturing the experience of emotions by athletes during competitive sport experiences. Jones et al. (2005) reached a consensus on 3 subscales to reflect unpleasant states associated with sport competition, inclusive of anger, anxiety, and dejection. There are 14 items that pertain to these 3 subscales. Four items for anger, five for anxiety and five for dejection.

### Data processing

Following the removal of individuals who provided incomplete data (*n* = 918), could not be placed according to a game genre (*n* = 13), or provided extreme responses (for instance answering 1 or 5 for every single item) (*n* = 20), data for 1,007 of the original 1958 participants were brought forward for analyses. Of the 1,007 participants, 797 reported as male, 173 female and 37 as nonbinary/did not disclose. Participants reported a mean age of 24.24 ± 6.20 (Mean ± SD), played video games an average of 15.91 h a week ±12.64, and had 11.94 ± 7.14 years of gaming experience. A comprehensive summary of participants’ details is provided in Table 1 according to the reported game genre played.

**Table 1:** Descriptives of gaming experience (mean ± SD) by game genre played.

Genre	Participants	Years gaming	Hours gaming/week
	(*N* = 1,007)	(Mean ± SD)	(Mean ± SD)
Real time strategy	72	13.40 ± 7.91	15.14 ± 11
Shooter games	361	10.63 ± 6.42	17.62 ± 13.37
MOBA	111	11.68 ± 7.13	18.26 ± 14
Sport games	77	10.75 ± 6.06	11.15 ± 9.85
Social simulation	24	11.21 ± 6.67	9.81 ± 8.47
Sim racing	35	11.34 ± 6.99	12.8 ± 9.82
Action adventure/RPG	301	13.64 ± 7.69	15.46 ± 12.64
Fighting	26	12.46 ± 7.95	13.7 3 ± 6.82

#### Data analyses

Data analyses were performed using SPSS version 28. Normality of data residuals was determined by observing Shapiro–Wilk statistics and examining histogram plots. Prior to conducting analyses that addressed the hypotheses of this study, we first sought to confirm the factor loading and reliability of the SEQ (Jones et al., 2005) and the BERQ (Kraaij and Garnefski, 2019) questionnaires for our target population as neither had previously been used in research pertaining to tilt. As the 2 items from the Severity of Tilting Scale were adapted for our study, reliability and factor loading were checked. A reliability analysis was conducted to determine the internal consistency of the scales and a factor analysis was conducted to determine how well the scale items represented the factors for our population of interest.

The SEQ questionnaire scale demonstrated high internal consistency (Cronbach’s α = 0.868). Additionally, indicators of factorability were good. The correlation matrix revealed evidence for moderate to high correlations, with no observable multicollinearity. The Kaiser-Mayer-Olkin test measures sampling adequacy and reported a value of 0.87, which is above the recommended value of 0.5, indicating the presence of a sufficient sample size. Bartlett’s test of sphericity demonstrated significance, meaning there is enough correlations between variables to conduct a factor analysis [χ^2^ (91) = 5929.374, *p* < 0.001]. Examination of the scree plot (Supplementary Figure S1), combined with consideration of eigenvalues above 1, and interpretation of the rotated factor matrix (Supplementary Table S1), all yielded support for the inclusion of all three factors. Inclusion of all 3 factors explained 62.02% of the variance in the data, with factor 1 (anxiety), 2 (anger) and 3 (dejection) accounting for 20.98, 20.88, and 20.17% of the variance following rotation, respectively.

The BERQ scale demonstrated adequate internal consistency (Cronbach’s α = 0.744). Additionally, indicators of factorability were good. The correlation matrix revealed evidence for moderate to high correlations, with no observable multicollinearity. The Kaiser-Mayer-Olkin test measures sampling adequacy and reported a value of 0.82, above the recommended value of 0.5, with Bartlett’s test of sphericity also demonstrated significance [χ^2^ (190) = 10999.69, *p* < 0.001]. Examination of the scree plot, combined with consideration of eigenvalues above 1, and interpretation of the rotated factor matrix, all yielded support for the inclusion of five factors. As observable in Supplementary Figure S2, the scree plot demonstrates that 5 factors explain 72.28% of the variance in the data, with factor 1 (withdrawal), factor 2 (actively approaching), factor 3 (social support), factor 4 (seeking distraction) and factor 5 (ignoring) accounting for 15.50, 15.22 14.31, 13.64, and 13.61% of the variance after rotation, respectively.

The two items adapted from the Severity of Tilting Scale demonstrated an internal consistency of (Cronbach’s α = 0.692). The correlation matrix revealed evidence for moderate correlations, with no observable multicollinearity. The Kaiser-Mayer-Olkin test measures sampling adequacy and reported a value of 0.50, meeting the recommended value of 0.5, indicating the presence of a sufficient sample size. Bartlett’s test of sphericity demonstrated significance [χ^2^ (1) = 329.526, *p* < 0.001]. Examination of the scree plot (Supplementary Figure S3), combined with consideration of eigenvalues above 1, yielded support for one factor. This factor explained 76.44.% of the variance in the data.

To address the first two hypotheses, which were to assess if there was a difference in experience of tilt based on (i) the reason to play and (ii) the gender of the player, a 2-way (Gender x Reason to Play) ANOVA based on tilt scores was conducted. To address our exploratory aim of identifying the influential factors that explain gamers’ experience of tilt, a multiple regression on tilt was conducted, with the age of player, hours spent playing, years of gaming experience, as well as scores from the behavioral emotion regulation and sport emotion questionnaire entered as predictor variables.

## Results

The first two hypotheses were concerned with determining if there was a difference in tilt based on the reason to play or gender. Data were normally distributed and displayed homogeneity of variance (Levene’s test *p* > 0.05). A two-way ANOVA demonstrated a significant effect of one’s reason to play on their experience of tilt [*F* (2, 998) = 8.882, *p* < 0.001, η^2^ = 0.017]. There was no significant main effect of gender [*F* (2, 998) = 0.059, *p* = 0.943, η2 = <0.001] and no interaction between reason to play and gender [*F* (4, 998) = 0.830, *p* = 0.056, η^2^ = 0.003].

**Figure 1:**
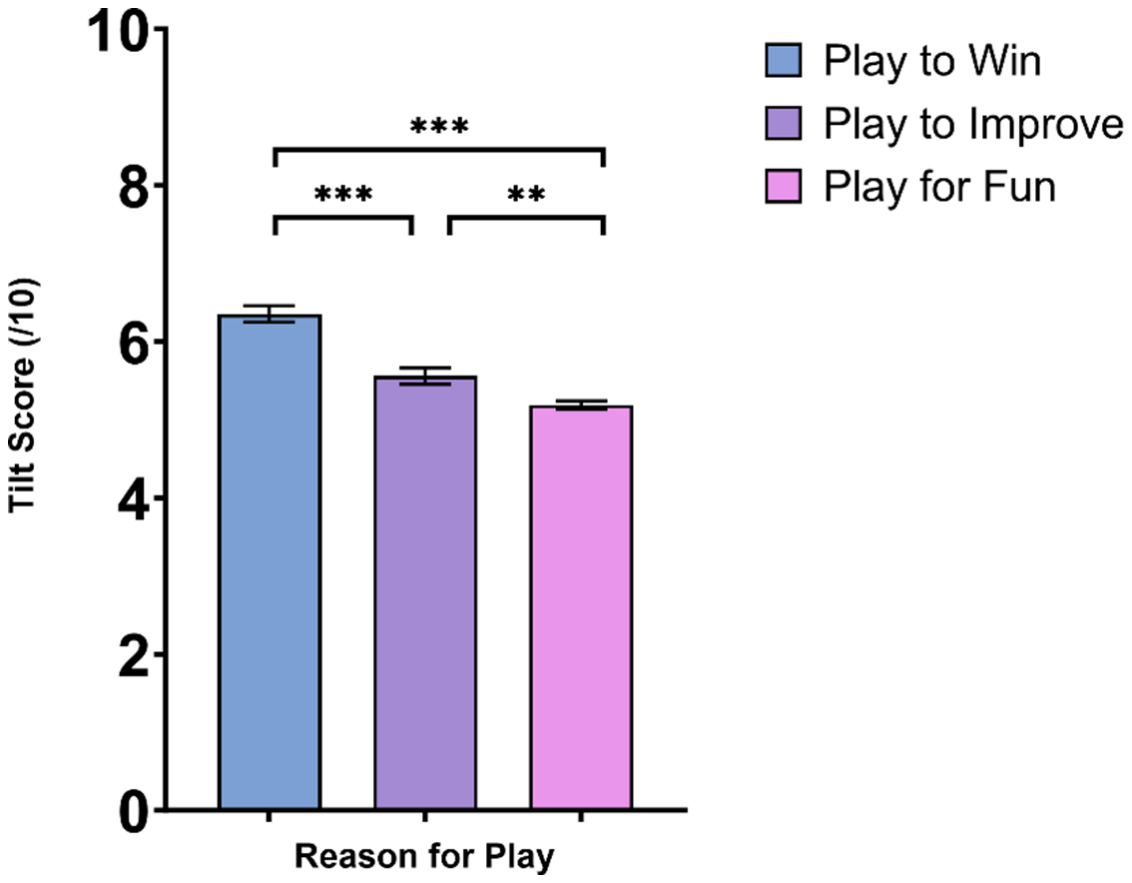
Experience of tilt severity by reason to play. ** and *** denote significance at *p* < 0.01 and *p* < 0.001, respectively. Figure denotes mean scores and standard error from the mean.

**Figure 2:**
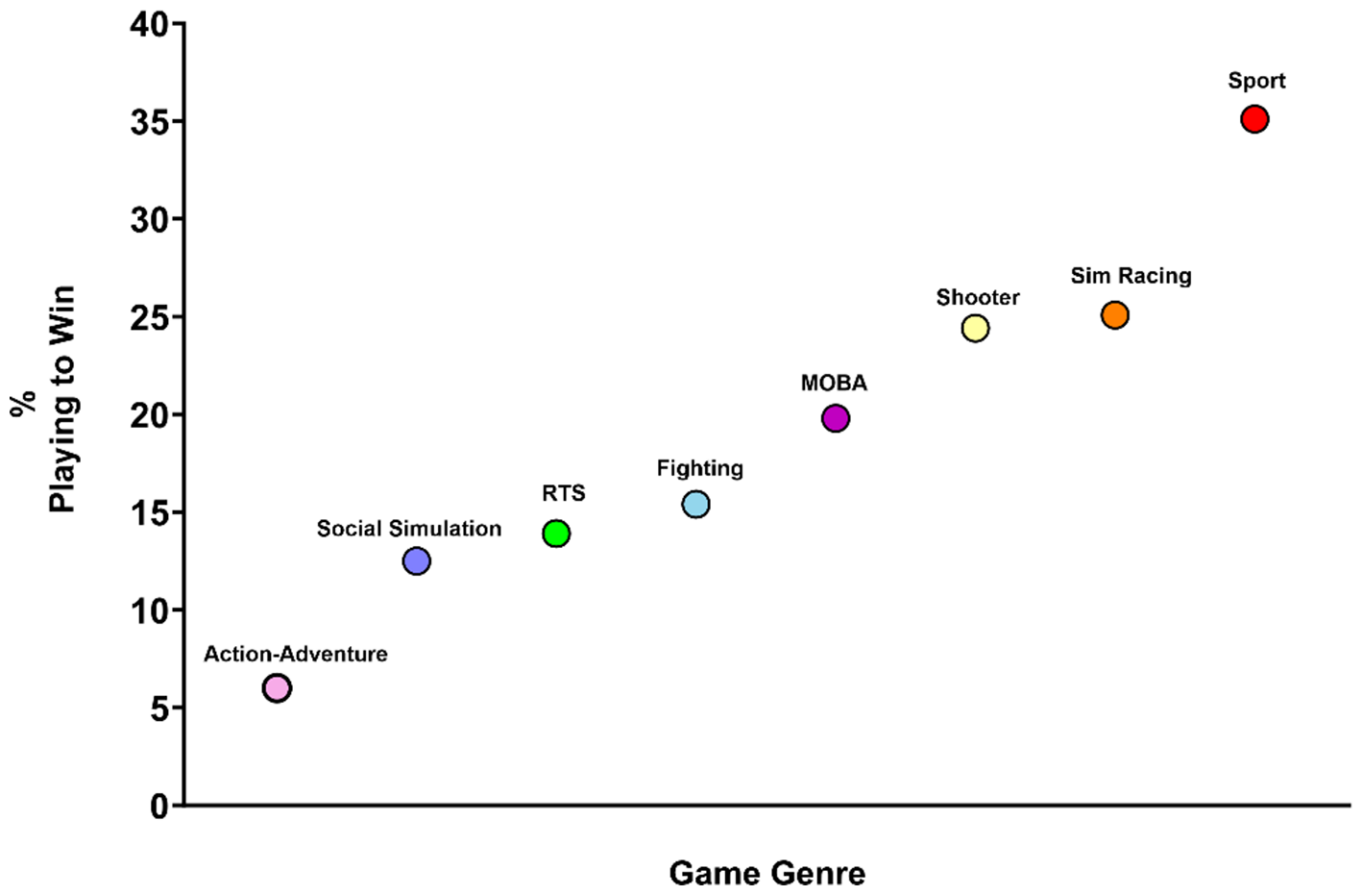
Percentage of players within each game genre playing to win.

*Post hoc* testing was conducted using Sidak alpha adjustments. Results indicated that experience of tilt was highest for those playing to win (Figure 1). Those that played to win had a significantly higher tilt score than those that played for fun (*p* < 0.001) and those that played to improve (*p* < 0.001). Those that played to improve had a significantly higher tilt score than those that played for fun (*p* = 0.006). A breakdown of the percentage of players from each game genre playing with the objective of winning is provided in Figure 2.

To address what factors most greatly influence one’s experience of tilt, a multiple regression was conducted with the gamers age, years gaming experience, hours spent gaming, BERQ and SEQ scores input as predictor variables. Assumptions were met to conduct a multiple regression. There was no multicollinearity in the data. Analysis of collinearity revealed variance inflation factor scores to be below 10, with statistics ranging from 1.062 to 1.724, respectively, and tolerance scores were well above 0.2, ranging from 0.580 to 0.941. Values of residuals were independent with Durbin-Watson statistic close to 2 (Durbin-Watson = 2.123). The P–P plot demonstrated that residuals were normally distributed. Furthermore, standardized residuals vs. standardized predicted values demonstrate no signs of funneling, indicative that the assumption of homoscedasticity has been met. No influential cases were found to bias the model with cook’s distance values all well under 1.

**Figure 3:**
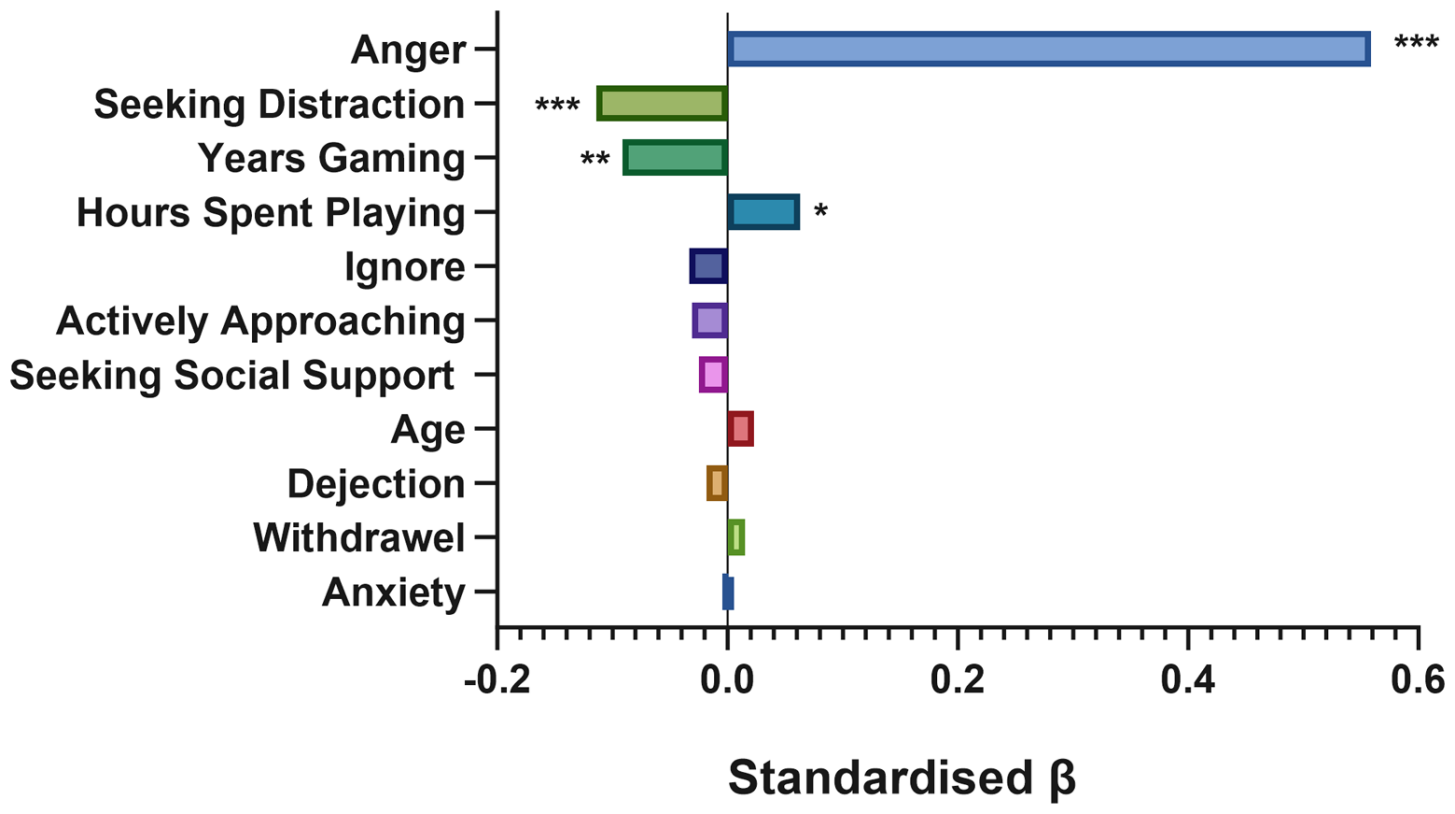
Standardized β’s of predictor variables entered in model. *,** and *** denote significant predictors of tilt severity at *p* < 0.05, *p* < 0.01 and *p* < 0.001, respectively.

Results from the multiple regression revealed that the predictor variables collectively had a significant effect on tilt score [*F* (11, 994) = 45.064, *p* < 0.001, *R*^2^ = 0.333]. Individual factors were examined revealing anger, seeking distraction, years spent gaming and hours spent gaming to be the significant predictors in the model (Table 2; Figure 3). As a result, the equation to predict tilt severity is:


$$\begin{array}{l} \mathrm{Tilt}\;\mathrm{Severity}\;\mathrm{Score}=\left(0.559\right)\;\mathrm{Anger}+\left(-0.114\right)\;\mathrm{Seeking}\;\mathrm{Distraction}+\\ \left(-0.091\right)\;\mathrm{Years}\;\mathrm{Gaming}+\\ \left(0.063\right)\;\mathrm{Hours}/\mathrm{Week}\;\mathrm{Gaming}+3.495 \end{array}$$

**Table 2:** Standardized β values and *p* values of predictor variables entered in model.

Predictor variables input	β	*p* value
Anger	0.559	<0.001***
Seeking distraction	−0.114	<0.001***
Years experience	−0.091	0.005**
Hours spent gaming	0.063	0.018*
Actively approaching	−0.031	0.255
Seeking social support	−0.025	0.383
Ignore	−0.033	0.237
Withdrawal	0.015	0.616
Age	0.023	0.486
Anxiety	0.001	0.983
Dejection	−0.018	0.605

*, ** and *** denote significant predictors of tilt severity at *p* < 0.05, *p* < 0.01 and *p* < 0.001, respectively. Dependent Variable = Tilt Score.

Anger and hours spent gaming were significant predictors for increasing likelihood of tilt. Years spent gaming and engagement in seeking distraction were significant predictors for decreasing likelihood of tilt. A summary of the results from the multiple regression are reported in Table 2, Figure 3, with standardized beta coefficients and *p* values.

As anger was identified as the most important predictor variable of tilt, a two-way ANOVA was conducted to determine if there were any differences in experience of anger based on the gender and genre. There was a significant effect on experience of anger based on gaming genre played [*F* (7, 986) = 2.757, *p* = 0.008, η^2^ = 0.019]. There was no significant effect based on gender [*F* (2, 986) = 0.483, *p* = 0.504, η^2^ = 0.001], nor was there a significant interaction between genre and gender [*F* (11, 986) = 0.583, *p* = 0.679, η^2^ = 0.008]. *Post hoc* testing was conducted using Sidak corrections and showed anger scores to be significantly higher for MOBA players compared to action-adventure players (*p* = 0.003).

As seeking distraction was another important predictor variable, a two-way ANOVA was also conducted to examine if there were any differences in seeking distraction scores based on game genre played by and the gender of gamers. No significant main effect was found for any of gaming genre played [*F* (7, 986) = 0.778, *p* = 0.606, η^2^ = 0.005] gender [*F* (2, 986) = 2.726, *p* = 0.066, η^2^ = 0.005], or the interaction between genre and gender [*F* (11, 986) = 1.000, *p* = 0.444, η^2^ = 0.011] on seeking distraction.

## Discussion

The current study aimed to examine gamers’ experience of tilt and emotion regulation. When examining reason for playing video games, we found that those playing for competitive reasons, were the most likely to experience tilt. The study also aimed to determine what factors were influential in gamers’ experience of tilt. Seeking distraction, anger, more years’ experience gaming and increased hours spent gaming were identified as important factors in predicting gamers’ experience of tilt.

In line with our first hypothesis, we found differences in the gamers experience of tilt based upon reason to play. Those motivated play to win were the most likely to experience tilt. As highlighted by Breuer et al. (2015) competition in games can be a source of frustration when a player cannot achieve their goal. Additionally, García-León et al. (2003) highlight that engagement in competitive tasks result in greater cardiovascular reactivity compared to engagement in problem solving tasks, illustrating the potential role of competition on physiological stress. Furthermore, in a study by Mendoza et al. (2021) examining esports competition, the researchers found competitive gaming context and player expertise impacted stress response.

In finding those play to win reporting higher levels of tilt, our study draws parallels with research on choking in traditional sport, whereby it is recognized that being highly motivated to win increase’s one’s susceptibility to choking (Beilock and Gray, 2007). Neuroscientific support for this is offered by Mobbs et al. (2009) who demonstrated via a functional magnetic resonance imaging (fMRI) study that the higher the reward for winning, the greater the likelihood of choking. Mobbs et al. (2009) revealed increased activity in reward regions as individuals got closer to winning (inclusive of the ventromedial striatum, dorsolateral striatum, right medial orbitofrontal cortex, and rostral anterior cingulate cortex) and, an association between increased midbrain activity and choking, evidenced in decrements in performance and increased errors by participants. Mobbs et al. (2009) also found differences between high and low reward conditions as distance to reward decreased, with increased activity observable in left ventral midbrain, including substantia nigra, right dorsolateral striatum and bilateral ventral premotor area in high reward condition compared to low reward condition, a finding which helps consolidate the reasoning that it is the competitive desire to win and potentially, the level of reward contributing to one’s experience of tilt.

Of notable interest is that of the game genres included, those playing sport games, were the most likely to play for competitive reasons, with 35% of sports players playing to win. It is plausible that those playing more competitive games, for more competitive reasons could be at increased susceptibility of experiencing tilt. Contrastingly, action-adventure players were the least likely to play for competitive reasons, with only 6% of action-adventure players playing to win, compared to 83.39% of action-adventure players playing for fun. As action-adventure games are focused upon immersing oneself in a story line (Pallavicini et al., 2021), it is plausible that the greater focus attributed to the story line encourages greater immersion and flow for players (Michailidis et al., 2018), offering a form of escapism (Prinsen and Schofield, 2021), with researchers recognizing that action-adventure games reduce anxiety and stress (Pallavicini et al., 2021). Granic et al. (2014) suggests playing games whereby an individual can switch between avatars with different skillsets, strengths, and vulnerabilities, facilitates flexibility in adjusting social and emotional goals. As a result, these types of games may promote flexible and adaptive emotion regulation in response to challenges.

The second hypothesis was that differences in tilt would exist based on gender. There were no significant differences found in experience of tilt based on gender. Research in traditional sport has examined choking under pressure during free throw performance (Toma, 2017). Given the similarities between choking we highlighted previously (Beilock and Gray, 2007; Mobbs et al., 2009), the findings from Toma (2017) can be seen to corroborate the results in this study and highlights the need for interventions centered upon improving emotion regulation to reduce tilt for all gamers.

This study also set out to examine which factors would be most influential in gamers experience of tilt. Seeking distraction, anger, years’ experience gaming and hours spent gaming were important predictive factors of one’s experience of tilt. Seeking distraction was identified as an important emotion regulation strategy to reduce an individual’s likelihood of experiencing tilt. An example of using this strategy included taking a break to engage in other enjoyable activities. Work by Waizman et al. (2023) supports the association between emotion regulation strategies and psychosocial responses to stress, with Kraaij and Garnefski (2019) and Leslie-Miller et al. (2023) suggesting seeking distraction to be a positive emotion regulation strategy to reduce stress and negative emotions. DiFrancisco-Donoghue and Jenny (2021) found that for esports players, taking breaks improved executive functioning among esports players, highlighting the potential benefit of break taking for gamer performance and experience.

Research in traditional sport also corroborates our finding that seeking distraction reduces one’s likelihood of experiencing tilt. For example, Balk et al. (2013) found distraction to be an adaptive emotion regulation strategy to alleviate choking and improve golf performance. Balk et al. (2013) suggest the possibility that it is the change in attentional focus brought by distraction that benefits performance, as attention is removed from the pressure of the event, reducing stress. A potential explanation for why distraction worked to reduce experience of tilt could be explained by explicit monitoring theory. This theory suggests that pressure raises self-consciousness among individuals, causing anxiety about performance (Beilock and Carr, 2001). The individual wants to perform well and so pays excessive attention to task execution, which disrupts automated skill execution, causing a usually automated process to become overly controlled (Baumeister, 1984; Yu, 2015). It is plausible that for the gamers, engagement in distraction is removing excessive attention away from the pressure of performance and therefore, reducing the experience of tilt.

Regarding the experience of negative emotions, anger was found to increase likelihood of experiencing tilt. MOBA players were found to experience higher anger than those playing action-adventure games in relation to tilt. A potential explanation for this could be attributed to toxicity observed in MOBA games, with Aguerri et al. (2023) finding that out of the 328 MOBA games they studied, 70% were disrupted by toxic behavior, most of which involved insults or complaining about teammate performance. Increased hours spent gaming also predicted one’s likelihood of experiencing tilt, with increased hours associated with an increased risk of experiencing tilt. Palomäki et al. (2014) study on poker corroborates with this, suggesting that playing more poker increases likelihood of experiencing tilt. Researchers highlight how excessive time spent playing video games can be problematic, finding it to be linked with increased anxiety, and depression (Wenzel et al., 2009).

Years of experience gaming was a significant predictor in one’s experience of tilt, with more years of experience reducing one’s likelihood of experiencing tilt. This finding is in line with Palomäki et al. (2014) observation that players perceived they tilted less severely when they accumulated more poker experience. The role of experience in moderating stress responses is well documented, with Arora et al. (2010) noting lower stress responses and better performance in experienced surgeons compared to novice surgeons in stressful conditions.

An important limitation to note this is that the criterion validity of the measure of tilt requires examination. Currently, there is no gold standard measure of tilt in gaming. However, to our knowledge, this is the first study to quantify gamers experience of tilt in video games, providing an important starting point in understanding tilt in gaming. Additionally, our sample was drawn from a predominantly European sample. It would be worthwhile to investigate how tilt manifests across different cultures, as research suggests cultural differences in emotional arousal (Lim, 2016). As this study is centered upon self-reported data, it would be worthwhile to empirically test if different game genres impact psychophysiological responses differently. As video games are played while sitting in front of a computer screen, a laboratory setting presents as an ecologically valid environment in which to conduct this type of research.

To conclude, this study set out to examine tilt in gaming and determine what factors contribute to this experience. We identified that there are differences in experience of tilt based on reason to play. Further, we identified factors which may increase in tilt (inclusive of anger and more hours spent playing) and factors which may protect against tilt (inclusive of years gaming experience and engagement in adaptive emotion regulation strategies). In doing so, we highlight how individuals playing video games for more competitive reasons may be at higher risk of experiencing tilt, demonstrating the need for greater focus to be attributed to supporting competitive gamers, a finding which provides valuable information for both esports coaches and players. This study provides an important starting point for understanding how to assess one’s risk of experiencing tilt, demonstrating the potential utility of emotion regulation interventions to improve gamers experience and performance, and better equipping us in understanding how to support gamers to improve their emotion regulation during gameplay.

## Data availability statement

The raw data supporting the conclusions of this article will be made available by the authors, without undue reservation.

## Ethics statement

The studies involving humans were approved by Faculty of Education and Health Sciences Research Ethics Committee at the University of Limerick. The studies were conducted in accordance with the local legislation and institutional requirements. The participants provided their written informed consent to participate in this study.

## Author contributions

SC: Data Curation, Formal analysis, Methodology, Conceptualization, Investigation, Writing – original draft, Writing – review & editing. AT: Formal analysis, Supervision, Visualization, Methodology, Writing – original draft, Writing – review & editing. MC: Supervision, Formal analysis, Funding acquisition, Visualization, Methodology, Writing – original draft, Writing – review & editing.
