# Supplementary material for: Playing for keeps or just playing with emotion? Studying tilt and emotion regulation in video games
Source: Front Psychol. 2024 Apr 26;15:1385242. doi: 10.3389/fpsyg.2024.1385242 (PMC11082399; doi:10.3389/fpsyg.2024.1385242)
Supplement: Supplementary file 1 [file Data_Sheet_1.pdf]

Supplementary Figure 1: Result of Scree Plot for our study sample using the Sports Emotion Questionnaire (Jones et al., 2005) subscales for Anger, Anxiety and Dejection.

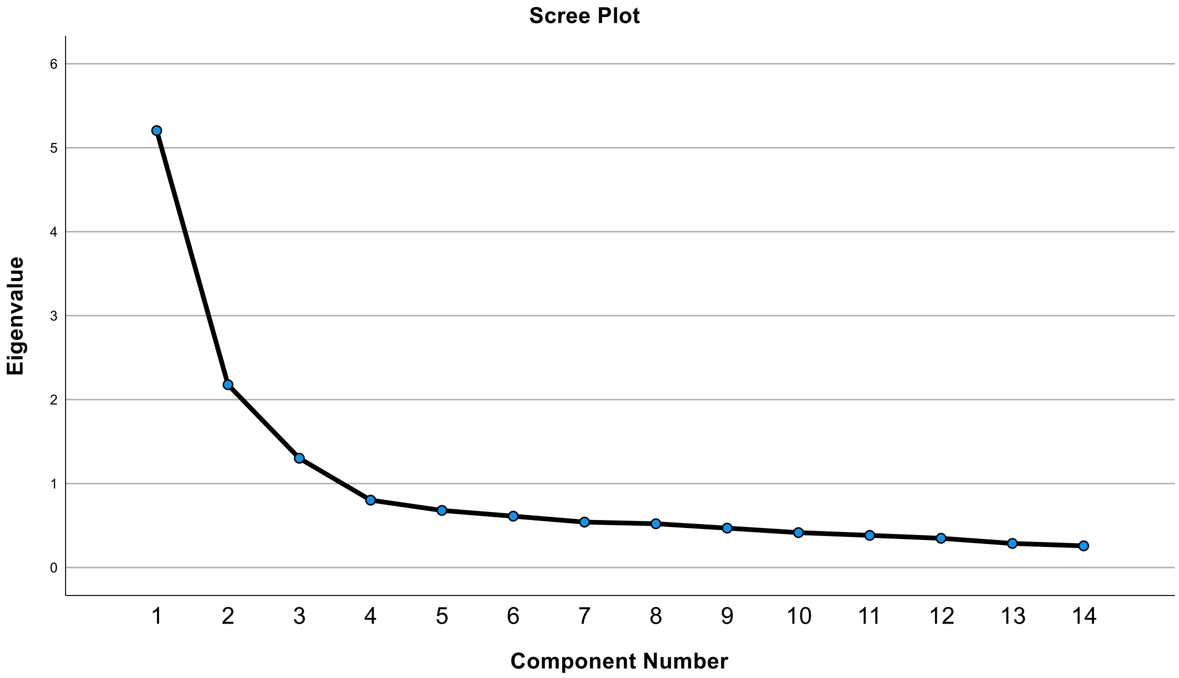

Supplementary Figure 2: Result of Scree Plot for our study sample using the Behavioural Emotion Regulation Questionnaire (Kraaij and Garnefski, 2019).

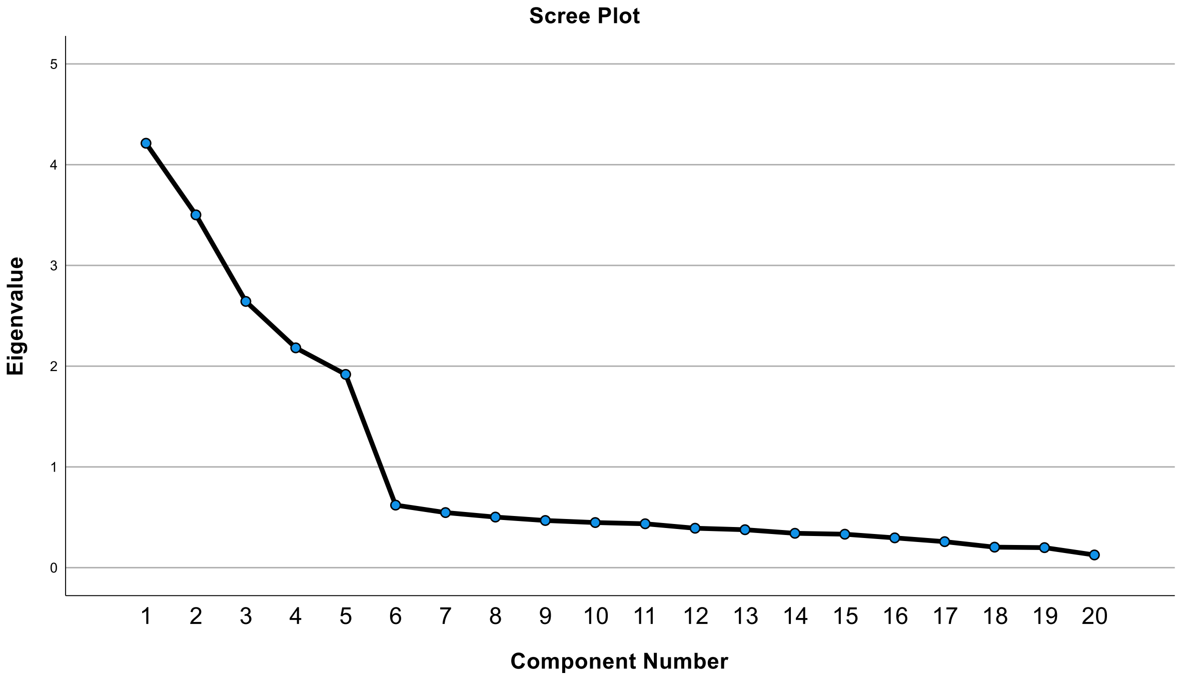

Supplementary Figure 3: Result of Scree Plot for our study sample using items adapted from the Severity of Tilting Scale **(**Palomäki et al., 2014)

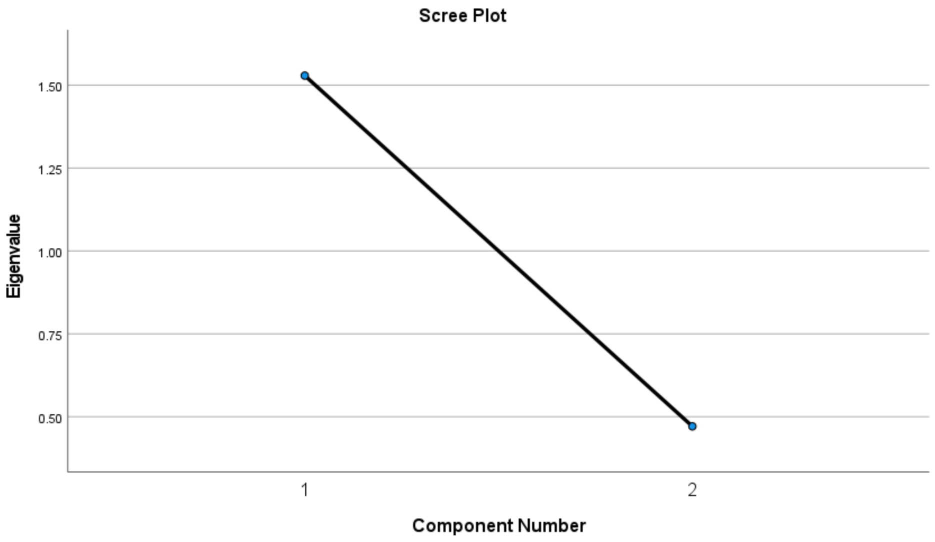

Supplementary Table 1: Results of the Rotated Factor Matrix Loadings for our study sample using the Sports Emotion Questionnaire (Jones et al., 2005) subscales for Anger, Anxiety and Dejection.

Factor structure of the SEQ prior to assignment to subscales

Scale name and items	*Factor Loadings*
**Anger**	
Annoyed	.773
Irritated	.808
Furious	.788
Anger	.800
**Anxiety**	
Nervous	.803
Anxious	.838
Tense	.629
Uneasy	.655

Apprehensive	.738
**Dejection**	
Unhappy	.632
Sad	.790
Upset	.693
Dejected	.716
Disappointed	.656

Supplementary Table 2: Results of the Rotated Factor Matrix Loadings for our study sample using the Behavioural Emotion Regulation Questionnaire (Kraaij and Garnefski, 2019).

Factor structure of the BERQ prior to assignment to subscales

Scale name and items	*Factor Loadings*
**Seeking Distraction**	
Engage in other, unrelated activities.	.775
Set my worries aside by doing something else.	.888
Do other things to distract myself.	.878
Engage in an activity which makes me feel good.	.705
**Withdrawal**	
Avoid other people.	.843
I withdraw.	.806
Isolate myself	.918

Close myself off to others.	.895
**Actively Approaching**	
Try do something about it.	.807
Get to work on it.	.887
Take action to deal with it.	.896
Do whatever is required to deal with it.	.825
**Seek Social Support**	
Look for someone to comfort me.	.819
Ask someone for advice.	.759
Share my feelings with someone.	.836
Look for someone who can support me.	.900
**Ignore**	
Move on and pretend nothing happened	.790
Repress it and pretend nothing happened	.808
Behave as if nothing is going on	.859
Block it Out	.802
